# The Minor Spliceosomal Protein U11/U12-31K Is an RNA Chaperone Crucial for U12 Intron Splicing and the Development of Dicot and Monocot Plants

**DOI:** 10.1371/journal.pone.0043707

**Published:** 2012-08-17

**Authors:** Kyung Jin Kwak, Hyun Ju Jung, Kwang Ho Lee, Young Soon Kim, Won Yong Kim, Sung Ju Ahn, Hunseung Kang

**Affiliations:** 1 Department of Plant Biotechnology and Kumho Life Science Laboratory, College of Agriculture and Life Sciences, Chonnam National University, Gwangju, Korea; 2 Department of Wood Science and Landscape Architecture, College of Agriculture and Life Sciences, Chonnam National University, Gwangju, Korea; 3 Bioenergy Research Center, College of Agriculture and Life Sciences, Chonnam National University, Gwangju, Korea; 4 Department of Bioenergy Science and Technology and Bioenergy Research Center, College of Agriculture and Life Sciences, Chonnam National University, Gwangju, Korea; Lehigh University, United States of America

## Abstract

U12 intron-specific spliceosomes contain U11 and U12 small nuclear ribonucleoproteins and mediate the removal of U12 introns from precursor-mRNAs. Among the several proteins unique to the U12-type spliceosomes, an *Arabidopsis thaliana* AtU11/U12-31K protein has been shown to be indispensible for proper U12 intron splicing and for normal growth and development of *Arabidopsis* plants. Here, we assessed the functional roles of the rice (*Oryza sativa*) OsU11/U12-31K protein in U12 intron splicing and development of plants. The *U11/U12-31K* transcripts were abundantly expressed in the shoot apical meristems (SAMs) of *Arabidopsis* and rice. Ectopic expression of OsU11/U12-31K in AtU11/U12-31K-defecient *Arabidopsis* mutant complemented the incorrect U12 intron splicing and abnormal development phenotypes of the *Arabidopsis* mutant plants. Impaired cell division activity in the SAMs and inflorescence stems observed in the AtU11/U12-31K-deficient mutant was completely recovered to normal by the expression of OsU11/U12-31K. Similar to *Arabidopsis* AtU11/U12-31K, rice OsU11/U12-31K was determined to harbor RNA chaperone activity. Collectively, the present findings provide evidence for the emerging idea that the U11/U12-31K protein is an indispensible RNA chaperone that functions in U12 intron splicing and is necessary for normal development of monocotyledonous plants as well as dicotyledonous plants.

## Introduction

Splicing of both the major class of U2-dependent introns and minor class of U12-dependent introns is an indispensible step in the regulation of gene expression in eukaryotes. Although U12-type minor introns, which constitute less than 0.5% of all introns in animals and plants [1]–[4], are significantly less frequent than U2-type major introns, their importance is substantial in the growth and development of animals and plants. The roles of U12 intron-specific small nuclear RNAs (snRNAs) in U12 intron splicing and the development of *Drosophila*, zebra fish, and human have been demonstrated [5]–[9], and the roles of U11/U12-specific proteins in cell viability and nonsense-mediated mRNA decay have also been demonstrated [2], [10], [11].

Splicing of the minor class of U12-type introns and major class of U2-type introns is catalyzed by the minor spliceosome and major spliceosome, respectively [2], [12]–[14]. During minor spliceosome formation, the U11 and U12 small nuclear ribonucleoproteins (snRNPs) form a stable U11/U12 di-snRNP complex [15], [16], and seven unique proteins, denoted 65K, 59K, 48K, 35K, 31K, 25K and 20K, specifically associate with the U11/U12 di-snRNPs [11], [17]. These minor spliceosome-associated proteins were found to be well conserved in animals and plants, and in plants they are conserved in both dicotyledonous and monocotyledonous plants [11], [18], [19].

Despite the well-conserved sequences of the minor spliceosome-associated proteins in animals and plants, the significance and biological roles of many minor spliceosomal proteins have not yet been experimentally demonstrated. It has been determined that U11/U12-65K, U11/U12-59K, and U11/U12-48K proteins are integral proteins in animals necessary for 5′ splice site selection and branchpoint site selection [20], [21]. Despite our increasing understanding of the roles of minor spliceosomal proteins in animals, reports demonstrating the functional roles of minor spliceosomal proteins in plants are severely limited. *Arabidopsis* AtU11/U12-35K was shown to facilitate recognition of the 5′ splice site [22]. In a previous study, we reported that *Arabidopsis* AtU11/U12-31K was essential for U12-type intron splicing by functioning as an RNA chaperone, and the AtU11/U12-31K-dependent U12 splicing was shown to be critical for normal plant development [23]. U11/U12-31K proteins are highly conserved in animals and plants, and in plants, the sequence of the U11/U12-31K proteins are well conserved in both dicotyledonous and monocotyledonous plants [18], [23], suggesting that they perform important cellular functions in plants. Considering our limited understanding of the roles of the minor spliceosome-associated proteins including U11/U12-31K protein, it would be of interest to determine whether U11/U12-31K proteins perform a similar function in monocotyledonous plants as well as in dicotyledonous plants. Here, we assessed the functional roles of the rice (*Oryza sativa*) OsU11/U12-31K protein in U12 intron splicing and the development of plants. By functional complementation of OsU11/U12-31K in the AtU11/U12-31K-defecient *Arabidopsis* mutant plants, we provide evidence demonstrating that U11/U12-31K proteins are functionally conserved RNA chaperones in *Arabidopsis thaliana* and rice, which are crucial for correct U12 intron splicing and normal development of monocotyledonous plants as well as dicotyledonous plants.

## Results

### Expression of *U11/U12-31K* in *Arabidopsis* and rice

**Figure 1:**
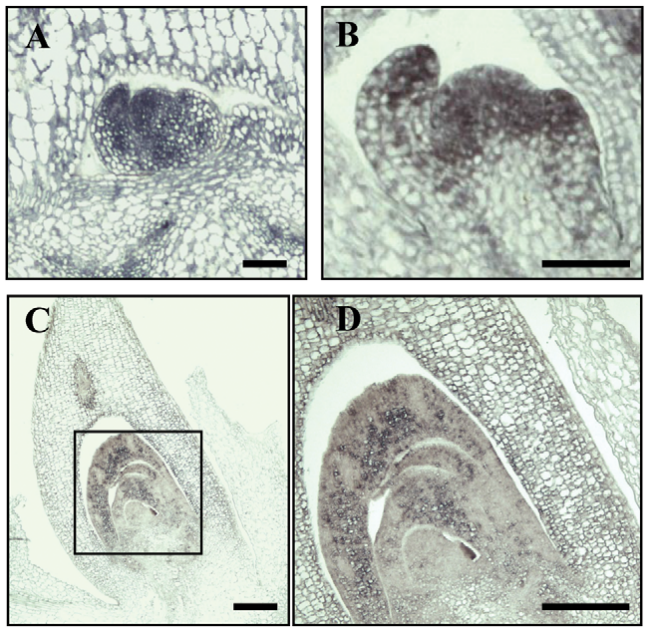
Expression patterns of *U11/U12–31K* in *Arabidopsis* and rice. Expression patterns of *U11/U12-31K* in (A, B) Arabidopsis 20 days after germination and (C, D) rice 3 days after germination as analyzed by *in situ* hybridization. Scale bar  = 50 µm.

Plant U11/U12-31K proteins are well conserved between dicotyledonous plants and monocotyledonous plants, with over 60 to 80% amino acid sequence identity [23]. In our previous analysis, *AtU11/U12-31K* was determined to be ubiquitously expressed in all *Arabidopsis* organs, including rosette leaves, cauline leaves, inflorescence stem, floral buds, and flowers [23]. Since AtU11/U12-31K affected the formation of primary inflorescence stems and meristem activity, we investigated in more detail the expression patterns of the *U11/U12-31K* gene in *Arabidopsis* and rice by *in situ* hybridization analysis. Results showed that *U11/U12-31K* was abundantly expressed in the shoot apical meristem (SAM) of *Arabidopsis* and rice (Figure 1). In addition, *U11/U12-31K* was abundantly expressed in elongating young leaves surrounding the SAM, but weakly expressed in mature leaves.

### U11/U12-31K is crucial for normal plant development

**Figure 2:**
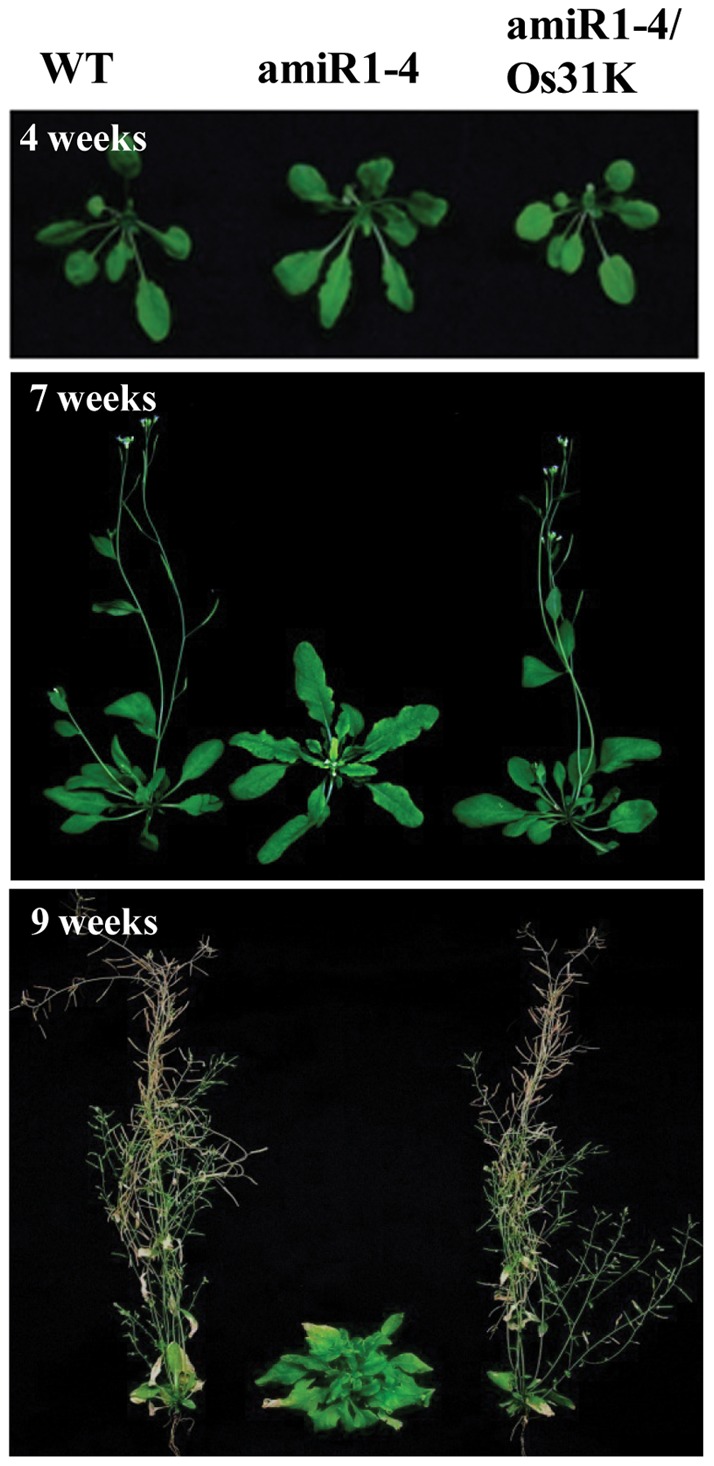
Recovery of normal phenotype of the mutant via OsU11/U12-31K complementation. The phenotypes of wild type (WT), AtU11/U12-31K knockdown mutant (amiR1-4), and complementation line expressing OsU11/U12-31K (amiR1-4/Os31K) were observed at different growth stages.

**Figure 3:**
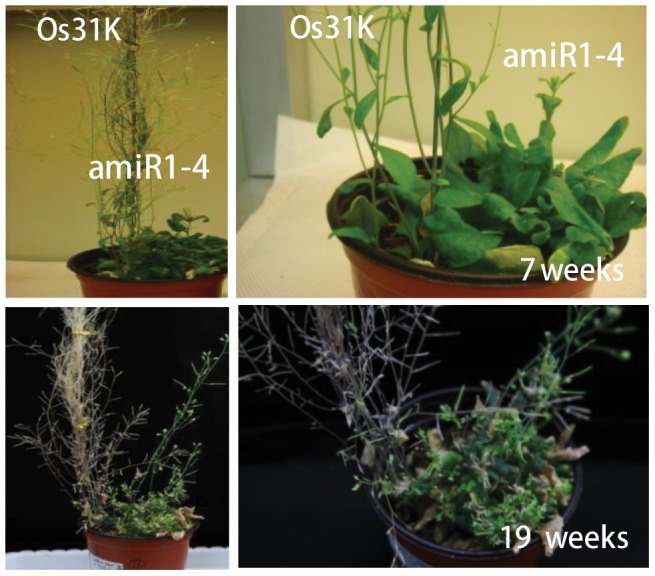
Maintenance of the prolonged cell division activity of AtU11/U12-31K knockdown plants. The knockdown plants (amiR1-4) survived beyond the death of the complementation lines expressing OsU11/U12-31K gene (Os31K).

Based on our previous observation that *Arabidopsis* AtU11/U12-31K plays an essential role in plant development [23], we wanted to determine whether rice OsU11/U12-31K is also crucial for plant development. To facilitate the functional analysis of OsU11/U12-31K, we used an artificial microRNA (amiRNA)-mediated AtU11/U12-31K knockdown plant. It has been demonstrated that amiR1-4, the hypermorphic allele of amiRNA-mediated AtU11/U12-31K knockdown plant, produces severely arrested primary inflorescence stems [23]. Therefore, we investigated the role of OsU11/U12-31K via functional complementation in the amiR1-4 mutant. Several independent transgenic lines expressing OsU11/U12-31K in an amiR1-4 background were generated, and their phenotypes were analyzed. The amiR1-4 mutant expressing OsU11/U12-31K showed wild type phenotypes (Figure 2). It is noteworthy that the degree of recovery was dependent on the levels of the *OsU11/U12-31K* transcript in the amiR1-4 mutant. The findings that OsU11/U12-31K successfully complemented the development-defect phenotypes of amiR1-4 mutant clearly demonstrated that the function of U11/U12-31K is conserved in *Arabidopsis* and rice. Although the amiR1-4 mutant exhibited severe defects in the formation of primary inflorescence stems, the knockdown plants survived beyond the death of the wild-type and complementation lines expressing OsU11/U12-31K gene (Figure 3). These results indicate that prolonged cell division activity is maintained in AtU11/U12-31K knockdown plants when compared to the wild-type plants.

**Figure 4:**
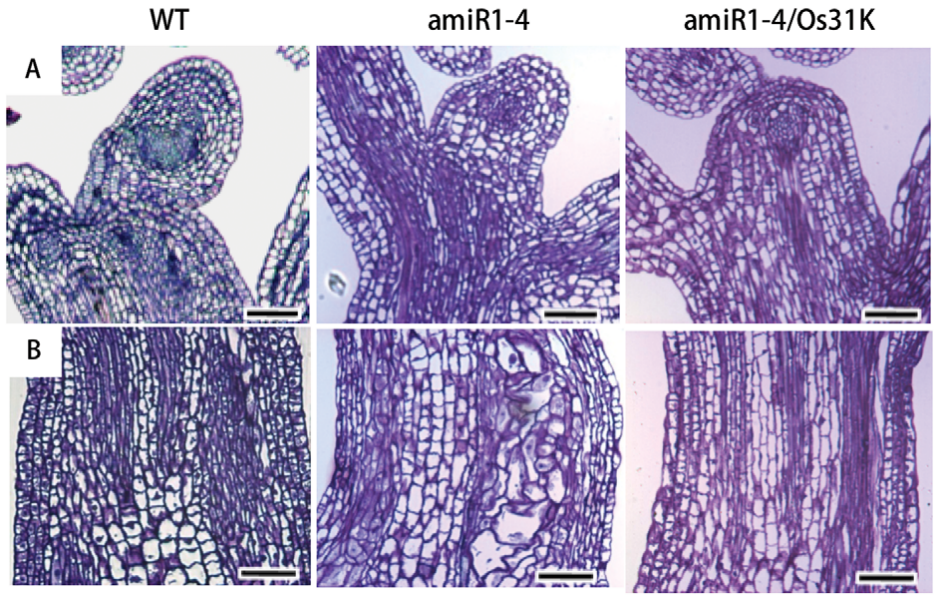
Images of the shoot apical meristem and the stem regions of the *Arabidopsis* plants. (A) Longitudinal sections of the SAMs of the 4-week-old wild type (WT), AtU11/U12-31K knockdown mutant (amiR1-4), and complementation line expressing OsU11/U12-31K (amiR1-4/Os31K). Bar  = 50 µm. (B) Longitudinal sections through the stems of 4-week-old WT, amIR1-4, and amiR1-4/Os31K plants. Bar  = 50 µm.

To better understand the cellular role of U11/U12-31K in regulating plant development, the morphologies of the vegetative SAM and the inflorescence stems were examined in 4-week-old wild-type, miR1-4 mutant, and miR1-4/Os31K complementation plants. The typical regular structure and organization of the SAM were maintained in miR1-4 mutant plants as well as in the wild-type and complementation plants, but the cell division activity in the SAM was diminished in the mutant plants (Figure 4A). However, the structure and shape of the cells in the inflorescence stems was significantly altered in the miR1-4 mutant, while the typical regular structure of cells was restored in the miR1-4/Os31K complementation plants (Figure 4B). The structures of the inflorescence and floral bud were also altered in the miR1-4 mutant plants compared with the wild-type plants (Figure S1). These abnormal morphologies of the miR1-4 mutant plants were completely recovered to normal structures in the miR1-4/Os31K complementation plants (Figure S1). All of these results indicate that U11/U12-31K plays a role in regulating cell division in the inflorescence stems, which is crucial for the normal development of monocot and dicot plants.

### Splicing defects in amiR1-4 mutant are rescued by the expression of OsU11/U12-31K

**Figure 5:**
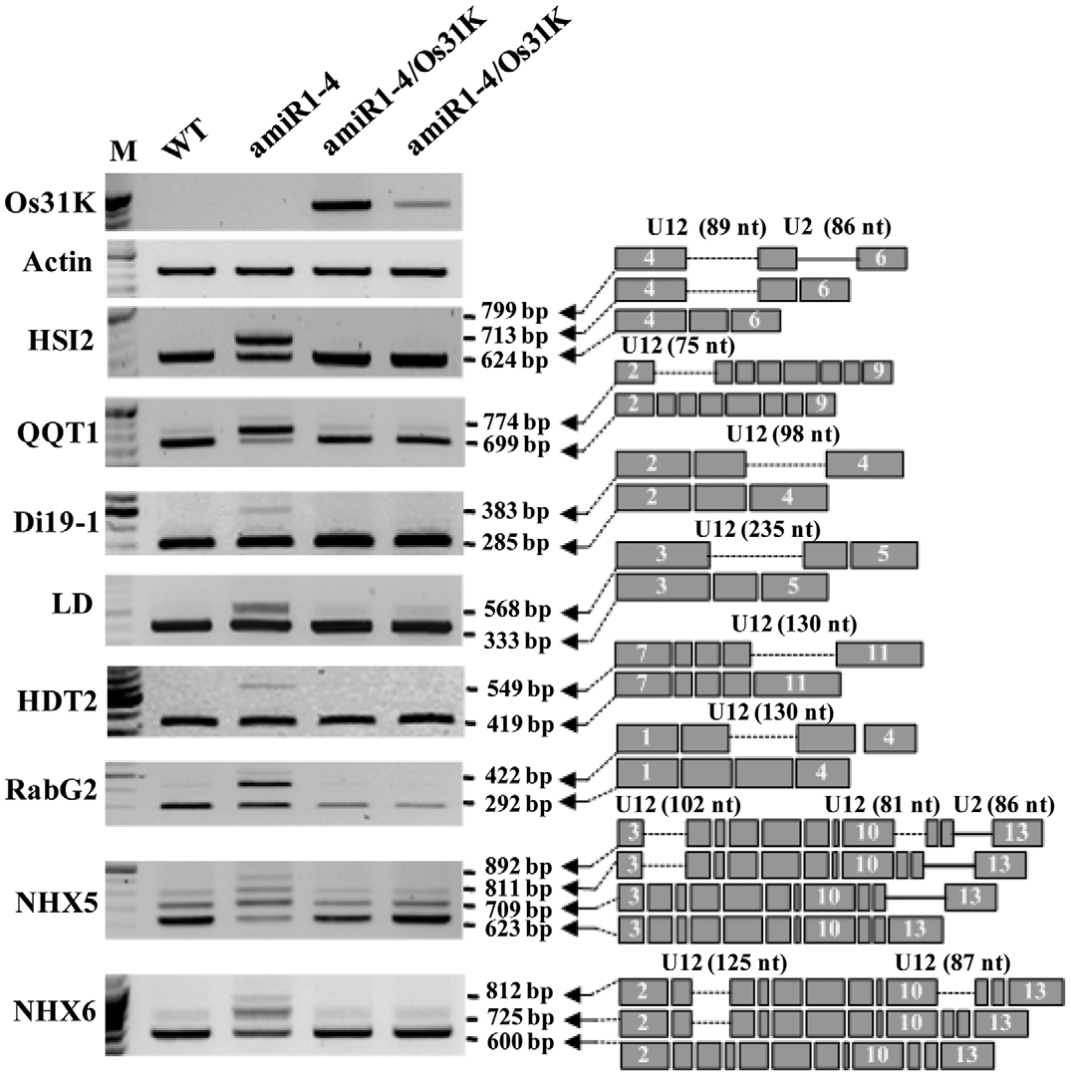
Recovery of normal splicing activity in the mutant via OsU11/U12-31K complementation. The splicing patterns of U12 intron-containing transcripts were analyzed by RT-PCR in wild type (WT), AtU11/U12-31K knockdown mutant (amiR1-4), and complementation lines expressing OsU11/U12-31K (amiR1-4/Os31K). Identical results were obtained from three independent experiments, one of which is shown. Expression of *OsU11/U12-31K* in each complementation line (amiR1-4/Os31K) was confirmed by RT-PCR.

To ascertain whether the recovery of normal phenotypes observed in the complementation lines was correlated with the maintenance of normal splicing of U12 introns by the expression of OsU11/U12-31K, we determined the splicing patterns of U12-type introns in the complementation plants as well as in wild type and mutant plants. Thirty U12 intron-containing genes out of the 165 U12-type intron-containing genes in *Arabidopsis* [3] were randomly selected and their splicing patterns analyzed (Table S1), as previously described [23]. The splicing of most U12 introns investigated in this study was defective in the amiR1-4 mutant plants, whereas the splicing of these U12 introns was normal in the complementation lines (Figure 5 and S2). These results indicate that the occurrence of normal splicing of U12 introns is correlated with the wild-type phenotypes of the complementation lines, and confirm that the function of U11/U12-31K in the process of U12 intron splicing is conserved in dicot and monocot plants.

**Figure 6:**
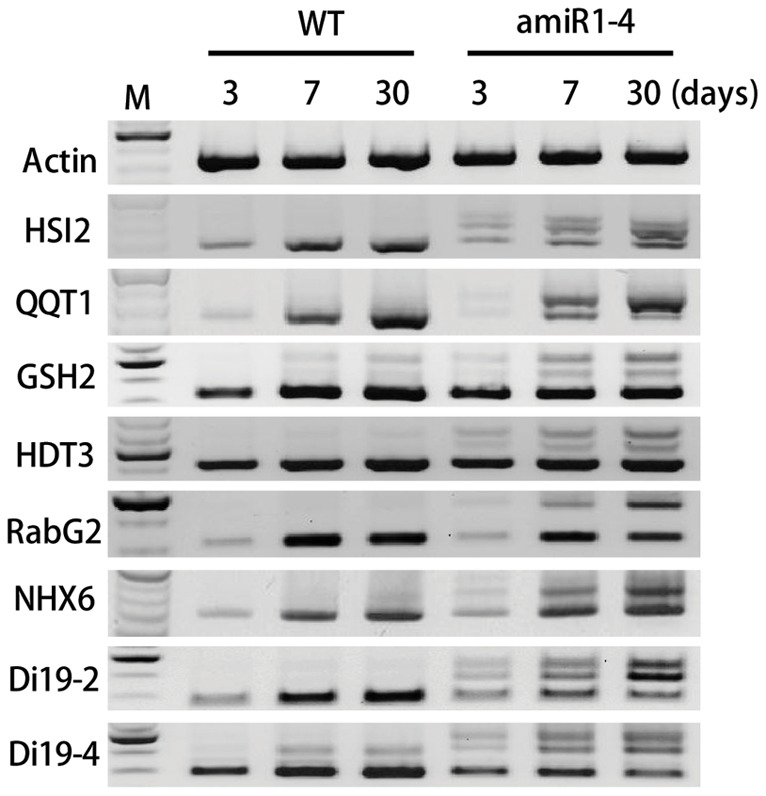
Development-dependent splicing of U12 intron-containing transcripts. The splicing patterns of U12 intron-containing transcripts were analyzed by RT-PCR in wild type (WT) and AtU11/U12-31K knockdown mutant (amiR1-4) at 3, 7, and 30 days after germination. Identical results were obtained from three independent experiments, one of which is shown.

Since miR1-4 mutant exhibited normal growth until bolting and showed severe defect in the formation of primary inflorescence stems, we investigated in more detail whether the splicing of U12 intron-containing genes is regulated by a developmental stage-dependent manner. The splicing patterns of U12 intron-containing transcripts in wild type and miR1-4 mutant plants were analyzed at 3, 7, and 30 days after germination (DAG) (Figure 6). Several transcripts, including HSI2, QQT1, RabG2, NHX6, and Di19-2, were expressed at low levels at 3 DAG but the expression of all transcripts at 7 and 30 DAG was similar to each other. Importantly, the splicing patterns of U12 intron transcripts in miR1-4 mutant at 7 DAG were similar to those at 30 DAG (Figure 6 and data not shown). These results indicate that the defect in U12 intron splicing does not affect the growth of *Arabidopsis* until bolting, which strengthen the notion that U11/U12-31K-directed splicing of U12 intron-containing genes influences the development of *Arabidopsis* specifically after bolting stage.

### Both *Arabidopsis* and rice U11/U12-31K proteins harbor an RNA chaperone activity

**Figure 7:**
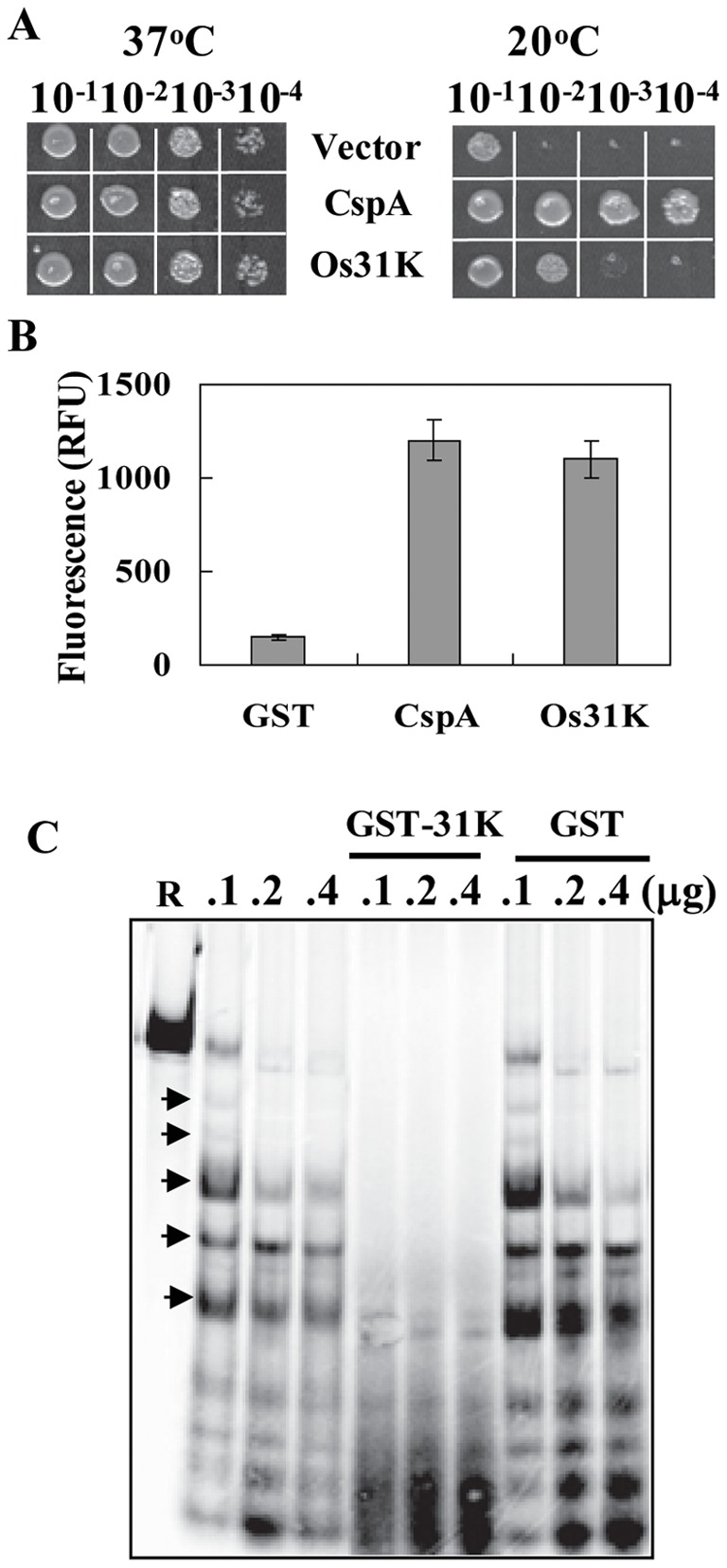
RNA chaperone activity of OsU11/U12-31K protein. (A) The colony-forming abilities of diluted BX04 *E. coli* cells expressing either OsU11/U12-31K (Os31K), CspA (positive control), or pINIII (negative control) were examined under normal (37°C) and cold stress (20°C) conditions. (B) The DNA-melting activity of OsU11/U12-31K protein was examined by monitoring the fluorescence of a 78-nucleotide-long molecular beacon with the addition of GST-Os31K (10 µg), GST-CspA (5 µg), or GST (5 µg). (C) Enhanced RNase T_1_ cleavage of the substrate RNA (R) was measured after the addition of OsU11/U12-31K protein (GST-31K). The RNase T_1_-resistant bands, as indicated by arrows, disappeared in the presence of recombinant GST-31K fusion protein. The numbers above each column represent the amount of T1 added to the reaction.

The findings that U11/U12-31K is involved in the splicing of most U12 intron-containing genes indicate that it may function as an RNA chaperone, which interact with diverse RNA substrates with low sequence specificity [24], [25]. In our previous report, *Arabidopsis* AtU11/U12-31K was shown to harbor RNA chaperone activity [23]. To determine whether rice OsU11/U12-31K also harbors RNA chaperone activity, several well-established methods were employed. First, the complementation ability of the OsU11/U12-31K protein was evaluated in cold-sensitive *E. coli* BX04 mutant cells, which lack four cold shock protein (CSP) genes that are bacterial RNA chaperones [26]–[28]. The BX04 cells expressing either OsU11/U12-31K, CspA (positive control), or pINIII vector (negative control) grew well under normal growth conditions (37°C) with no noticeable differences (Figure 7A). However, when subjected to low temperature (20°C), only the BX04 cells expressing either OsU11/U12-31K or CspA grew well, whereas the BX04 cells harboring the pINIII vector did not (Figure 7A). This complementation ability of OsU11/U12-31K in the cold sensitive BX04 cells suggests that it functions as an RNA chaperone. Secondly, the nucleic acid-melting ability of OsU11/U12-31K was evaluated to further demonstrate its RNA chaperone activity. Recombinant glutathione *S*-transferase (GST)-31K protein and GST-CspA protein were purified from *E. coli* (Figure S3) and utilized for nucleic acid-melting analysis. The DNA-melting ability of OsU11/U12-31K was evaluated by using a partially double-stranded DNA molecule that produces fluorescence signals upon melting. Strong fluorescent signals were observed upon the addition of recombinant GST-31K or GST-CspA (positive control), but not the addition of GST protein (negative control), to the reaction mixture (Figure 7B), indicating that OsU11/U12-31K possesses *in vitro* DNA-melting ability. We next performed the RNase T_1_ cleavage assay to determine whether OsU11/U12-31K is capable of melting RNA secondary structures. The addition of RNase T_1_ to the RNA substrates transcribed from the pET-22b(+) plasmid produced several RNase T_1_-resistant bands (Figure 7C, indicated by arrows). These RNase-resistant bands disappeared when recombinant GST-31K was added before RNase T_1_. However, cleavage of RNA substrates by RNase T_1_ was not affected by the addition of GST. These results indicate that OsU11/U12-31K destabilizes the base pairings in the RNA substrates, allowing RNase T_1_ to further digest RNAs into smaller fragments. All of these results clearly demonstrate that OsU11/U12-31K is capable of melting nucleic acids and harbors RNA chaperone activity.

## Discussion

The results of our study clearly indicate that the U11/U12-31K protein, one of the seven unique minor spliceosomal proteins, is a functionally conserved RNA chaperone, which are crucial for correct U12 intron splicing and normal development of monocotyledonous plants as well as dicotyledonous plants. The presence of a single gene encoding U11/U12-31K in plant genomes and the highly similar amino acid sequences of U11/U12-31K proteins in dicots and monocots have indicated that their function is conserved in plants. The functional complementation of OsU11/U12-31K in the *Arabidopsis* mutant diminishing the expression of *AtU11/U12-31K* clearly demonstrated that U11/U12-31K plays an essential role in the development of monocot as well as dicot plants. Future research in rice is needed to fully confirm that U11/U12-31K protein is necessary for the development of monocot plants. We observed a much higher expression of *U11/U12-31K* in the SAM of *Arabidopsis* and rice (Figure 1), which correlated with the observed phenotypic defects in knockdown plants exhibiting stunted inflorescence stems. In addition, the structure and shape of the cells in inflorescence stems of *U11/U12-31K* knockdown mutant plants were abnormal (Figure 4). These results indicate that U11/U12-31K affects plant development by regulating meristem activity and cell division activity.

U12 introns are often found in genes that perform a function in the regulation of cell proliferation, such as DNA replication/repair, RNA processing, and translation [12], [29]. In our previous analysis, AtU11/U12-31K was shown to be responsible for the correct splicing of most of the U12 intron-containing genes [23], including QQT1(encoding ATP/GTP binding protein), HSI2 (encoding B3 DNA-binding transcription factor), Di19 (encoding gene family implicated in stress and light signaling pathways), and NHX (encoding Na^+^/H^+^ antiporter), all of which are closely related to cell division, seed maturation, and seedling growth [30]–[33]. The role for U11/U12-31K in U12-type intron splicing in dicots and monocots was confirmed in the present analysis. Defects in the correct splicing of all U12 introns in *Arabidopsis* AtU11/U12-31K mutants were completely recovered in the complementation lines expressing rice OsU11/U12-31K (Figure 5). These results indicate that the activity of the U11/U12-31K protein in the splicing of most U12-type introns on minor spliceosomal complexes is absolutely conserved in dicots and monocots, which is crucial for the normal development of plants.

The proposition that U11/U12-31K protein functions as an RNA chaperone during U12 intron splicing was firmly supported by the current and previous analyses. Both OsU11/U12-31K and AtU11/U12-31K proteins have been determined to harbor RNA chaperone activity, demonstrated by the functional complementation of RNA chaperone-deficient *E. coli* mutant and nucleic acid-melting activity (Figure 7) [23]. The observation that U11/U12-31K influences the splicing of most U12-type introns is another indication that U11/U12-31K functions as an RNA chaperone, since RNA chaperones usually adopt highly disordered structures and bind their RNA substrates with low sequence specificity [24], [25], [34], [35]. It is likely that, as an RNA chaperone, U11/U12-31K maintains the pre-RNA substrates in splicing-competent conformations or facilitates the rearrangement of spliceosomal RNAs and/or mRNAs during the splicing process. The U11/U12-31K protein is not a splicing factor, and it remains uncertain whether U11/U12-31K directly binds to the RNA substrates or if additional protein factors are required. The presence of an RNA recognition motif and CCHC-type zinc knuckle domain in U11/U12-31K strongly suggests that it directly binds to the RNA substrates. All of these results led us to hypothesize that U11/U12-31K is an RNA chaperone involved in U12 intron splicing.

In conclusion, the present findings provide evidence for the emerging idea that U11/U12-31K is an indispensible RNA chaperone that functions in U12-type intron splicing and is necessary for the normal growth and development of dicot as well as monocot plants. Considering that the biological functions of most minor spliceosomal proteins have not yet been determined in plants and animals, our findings open new opportunities to further investigate the roles of other highly conserved minor spliceosomal proteins in U12 intron splicing and for the growth and development of plants and animals.

## Materials and Methods

### Plant materials and growth conditions

*A. thaliana* Columbia-0 ecotype was grown at 23°C under long day conditions (16-hr-light/8-hr-dark cycle). Plants were also grown in half-strength Murashige and Skoog (MS) medium containing 1% sucrose. The *AtU11/U12-31K* knockdown *Arabidopsis* mutant plants (amiR1-4), in which the expression of *AtU11/U12-31K* is downregulated by an artificial microRNA-mediated knockdown strategy (Web MicroRNA Designer; http://wmd3.weigelworld.org/), were described in our previous report [23]. To generate complementation lines, the pCambia3301 vector carrying the rice *OsU11/U12-31K* gene under the control of the cauliflower mosaic virus 35S promoter was introduced into the *Arabidopsis* amiR1-4 mutant by vacuum infiltration [36] using *Agrobacterium tumefaciens* GV3101. The T_3_ homozygous lines were selected and used for phenotype analysis.

### *In situ* hybridization and microscopy

To determine the expression patterns of *U11/U12-31K* in different organs and cells, *in situ* hybridization analysis was conducted using different tissues of Arabidopsis and rice. Plant samples were fixed with 4% paraformaldehyde in 50 mM sodium phosphate buffer (pH 7.0), embedded in paraffin, and sliced into thin sections. The RNA probes were synthesized *in vitro* using a digoxigenin RNA labeling kit (Roche Molecular Biochemicals, Mannheim, Germany). The probes were hybridized to the samples, and the signals were detected by chemiluminescence. For scanning electron microscopy (SEM) observation, the samples were fixed with a mixture of 2% glutaraldehyde and 2% paraformaldehyde, and were post-fixed with 1% osmium tetroxide in 50 mM cacodylate buffer (pH 7.2). After dehydrating the samples with a series of alcohols, the samples were dried with a HCP-2 critical point dryer (Hitach, Tokyo, Japan), coated with gold in a Emitec K550 ion sputter, and observed with a Hitachi S-2400 scanning electron microscope (Hitachi, Tokyo, Japan). For light microscope observation, the samples which were embedded in LR White resin were sectioned into thin sections, stained with 0.1% toluidine blue, and examined with a light microscope (Zeiss, Axiolba, Germany).

### Analysis of the splicing of U12 intron-containing genes

For the analysis of splicing patterns of U12 intron-containing genes, total RNAs were extracted from 20-day-old wild-type, AtU11/U12-31K knockdown *Arabidopsis* mutant (amiR1-4), and OsU11/U12-31K-expressing amiR1-4 plants. Five to ten micrograms of total RNAs were treated with RQ1 DNase (Promega, Madison, WI, USA) and further purified using an RNeasy clean-up kit (Qiagen, Valencia, CA, USA). RT-PCR analysis of the splicing patterns was conducted as described previously [23]. Briefly, two hundred nanograms of RNAs were reverse-transcribed with gene-specific primers [23] and amplified using a one-step RT-PCR kit (Qiagen). The PCR products were separated on 1% agarose gel and visualized under UV light.

### Analysis of RNA chaperone activity

In the cold shock assay using the *E. coli* BX04 mutant cells, OsU11/U12-31K cDNA was cloned into the pINIII vector. The cold shock test for *E. coli* was conducted as described previously [23], [35]. The pINIII expression vector was transformed into *E. coli* BX04 mutant cells, and the growth of cells was monitored in Luria-Bertani (LB) medium at low temperatures. In the nucleic acid-melting assay, the 78-nucleotide-long hairpin-shaped DNA molecule labeled with a fluorophore (tetramethyl rhodamine) and quencher (dabcyl) was synthesized as described previously [28], and nucleic acid-melting assay was conducted according to our previous studies [23], [35]. The fluorescence emitted from the molecular beacon after reaction with recombinant GST-31K fusion proteins was measured using a Spectra Max GeminiXS spectrofluorometer (Molecular Devices, Sunnyvale, CA, USA) at an excitation and emission wavelengths of 555 and 575 nm, respectively. In ribonuclease cleavage assay, the ^32^P-labeled RNA substrates were prepared by transcription of the pET-22b(+) plasmid using T7 RNA polymerse (Promega). The RNA substrates were incubated with recombinant GST-31K fusion proteins, and the reaction products were separated on an 8% acrylamide gel. All experimental conditions were maintained as described previously [23], [35].

## Supporting Information

Figure S1 **Morphology of inflorescence stems of the plants.** (A) Light micrographs of floral bud regions of 7-week-old wild-type (WT), knockdown mutant (amiR1-4), and complementation line expressing OsU11/U12-31K gene (amiR1-4/Os31K). Scale bar  = 1mm. (B) SEM of inflorescence stems of 7-week-old wild-type, mutant, and complementation line.
(TIF)

Figure S2 **Abnormal splicing patterns of U12-type introns in the amiR1-4 mutant and complementation lines.** The splicing patterns of several U12 intron-containing transcripts were analyzed by RT-PCR in wild-type (WT), knockdown plants (amiR1-4), and complementation lines expressing OsU11/U12-31K gene (amiR1-4/Os31K). The experiment was repeated three times using different batches of RNA samples, and similar results were obtained. The gray boxes with numbers represent exons, and the dashed and solid lines represent U12 and U2 introns, respectively.
(TIF)

Figure S3 **Purification of GST-31K fusion proteins.** The recombinant GST fusion proteins were purified in *E. coli* and the purified GST, GST-CspA and GST-Os31K fusion proteins were analyzed by SDS-PAGE.
(TIF)

Table S1 **List of U12 intron-containing genes investigated in this study and their splicing patterns in the amiR1-4 mutant plant.**
(RTF)
