# Supplementary figures and images for: The Minor Spliceosomal Protein U11/U12-31K Is an RNA Chaperone Crucial for U12 Intron Splicing and the Development of Dicot and Monocot Plants
Source: PLoS One. 2012 Aug 17;7(8):e43707. doi: 10.1371/journal.pone.0043707 (PMC3422263; doi:10.1371/journal.pone.0043707)

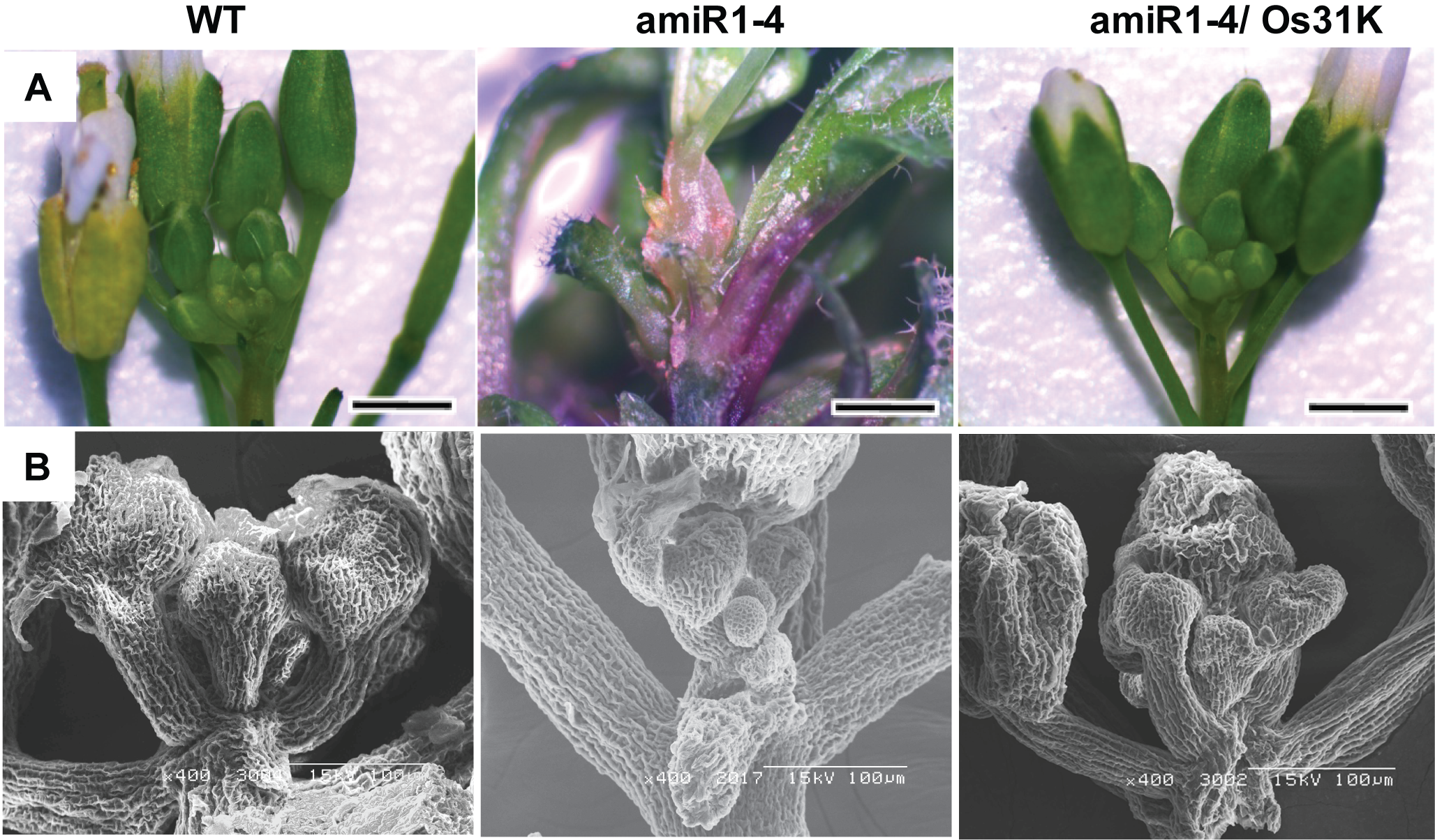

Supplement: Figure S1 — Morphology of inflorescence stems of the plants. (A) Light micrographs of floral bud regions of 7-week-old wild-type (WT), knockdown mutant (amiR1-4), and complementation line expressing OsU11/U12-31K gene (amiR1-4/Os31K). Scale bar = 1mm. (B) SEM of inflorescence stems of 7-week-old wild-type, mutant, and complementation line. (TIF) [file pone.0043707.s001.tif]

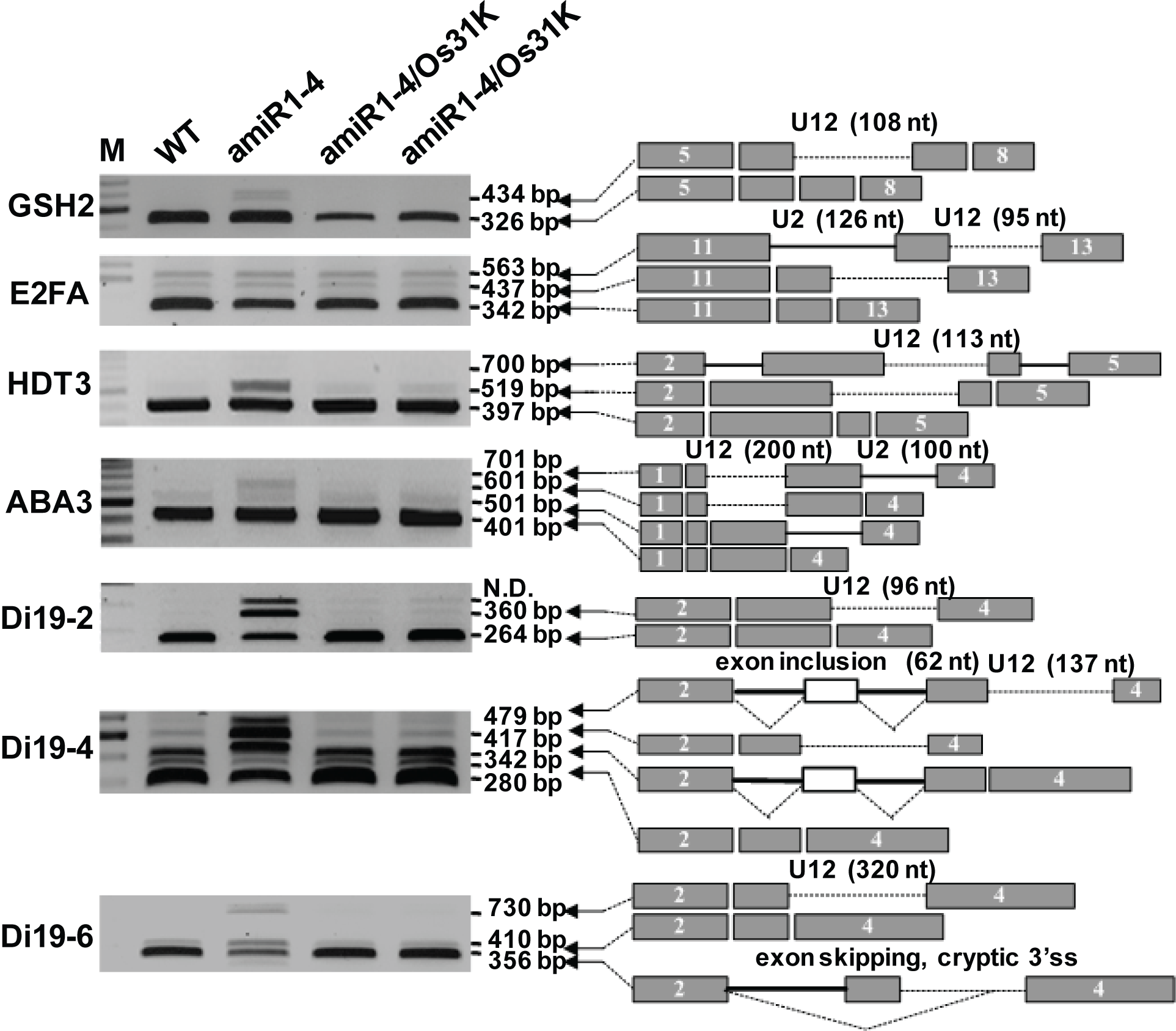

Supplement: Figure S2 — Abnormal splicing patterns of U12-type introns in the amiR1-4 mutant and complementation lines. The splicing patterns of several U12 intron-containing transcripts were analyzed by RT-PCR in wild-type (WT), knockdown plants (amiR1-4), and complementation lines expressing OsU11/U12-31K gene (amiR1-4/Os31K). The experiment was repeated three times using different batches of RNA samples, and similar results were obtained. The gray boxes with numbers represent exons, and the dashed and solid lines represent U12 and U2 introns, respectively. (TIF) [file pone.0043707.s002.tif]

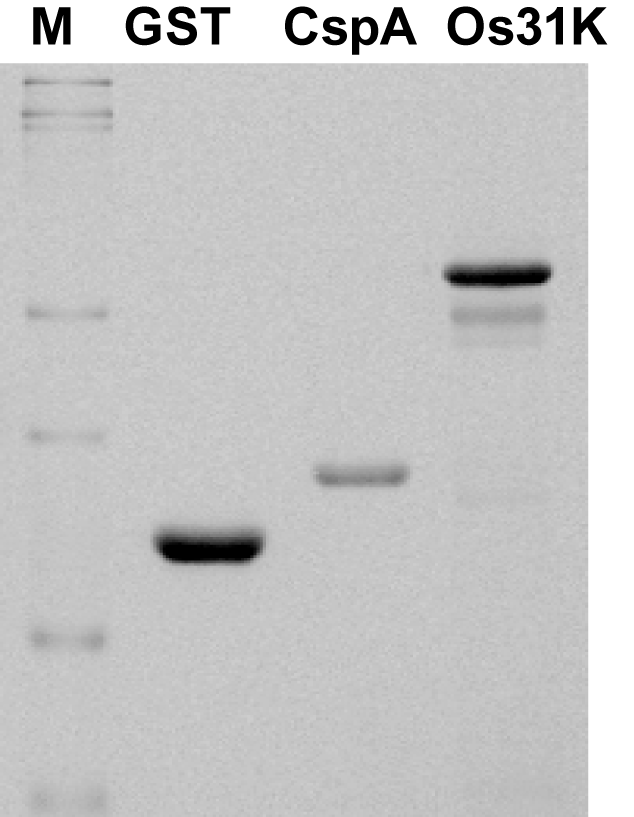

Supplement: Figure S3 — Purification of GST-31K fusion proteins. The recombinant GST fusion proteins were purified in E. coli and the purified GST, GST-CspA and GST-Os31K fusion proteins were analyzed by SDS-PAGE. (TIF) [file pone.0043707.s003.tif]
