# Supplementary material for: The Minor Spliceosomal Protein U11/U12-31K Is an RNA Chaperone Crucial for U12 Intron Splicing and the Development of Dicot and Monocot Plants
Source: PLoS One. 2012 Aug 17;7(8):e43707. doi: 10.1371/journal.pone.0043707 (PMC3422263; doi:10.1371/journal.pone.0043707)
Supplement: Table S1 — List of U12 intron-containing genes investigated in this study and their splicing patterns in the amiR1-4 mutant plant. (RTF) [file pone.0043707.s004.rtf]

Table S1. List of U12 intron-containing genes investigated in this study and their splicing patterns in the amiR1-4 mutant plants.

Gene	Accession no.	Putative function	Splicing affected	
QQT1	AT5G22370	ATP-binding family protein	yes	
LD	AT4G02560	Transcriptional regulator	yes	
NHX5	AT1G54370	Na+/H+ antiporter	yes	
NHX6	AT1G79610	Na+/H+ antiporter	yes	
GSH2	AT5G27380	Glutathione synthetase	yes	
HDT2	AT5G22650	Histone deacetylase-like protein	yes	
HDT3	AT5G03740	HD2-type histone deacetylase HDAC	yes	
RABG2	AT2G21880	Ras family GTP-binding protein	yes	
E2FA	AT2G36010	E2FA/E2F3 transcription factor	yes	
E2FB	AT5G22220	E2FA/E2F3 transcription factor	no	
ABA3 	AT1G16540	Molybdenum cofactor sulfurase	yes	
HSI2	AT2G30470	ABI3 family, B3 transcriptional repressor	yes	
CBP20	AT5G44200	Cap-binding protein	yes	
Di19-1	AT1G56280	CCHH-type zinc finger protein family	yes	
Di19-2	AT1G02750	Dehydration-induced 19 family	yes	
Di19-3	AT3G05700	Dehydration-induced 19 family	yes	
Di19-4	AT3G06760	Dehydration-induced 19 family	yes	
Di19-5	AT4G02200	Dehydration-induced 19 family	no	
Di19-6	AT5G26990	Dehydration-induced 19 family	yes	
Di19-7	AT5G49230	Dehydration-induced 19 family	yes	
ASHR3	AT4G30860	trxG protein family	no	
DCP2 	AT5G13570	mRNA decapping activity	yes	
NRPA2	AT1G29940	Subunit of RNA polymerase1	yes	
LIG4	AT5G57160	DNA ligase IV	yes	
WNK6	AT3G18750	WNK family of protein kinase	no	
RAD9	AT3G05480	Regulation of DNA damage repair	no	
U12-1	AT3G46210	Ribosomal protein S5 domain 2-like	yes	
U12-2	AT5G08500	Transmembrane CLPTM1 family protein	no	
U12-3	AT1G26660	Prefoldin chaperone subunit family protein	yes	
